# Early clinical response and presence of viral resistant minority variants: a proof of concept study

**DOI:** 10.7448/IAS.17.4.19759

**Published:** 2014-11-02

**Authors:** Annapaola Callegaro, Elisa Di Filippo, Noemi Astuti, Paula Andrea Serna Ortega, Marco Rizzi, Claudio Farina, Daniela Valenti, Franco Maggiolo

**Affiliations:** 1 Microbiology and Virology, AO Papa Giovanni XXIII, Bergamo, Italy; 2 USC Infectious Diseases, AO Papa Giovanni XXIII, Bergamo, Italy

## Abstract

**Introduction:**

Traditional genotyping assays detect viral variants present in at least 15–25% of the entire virus population. We tested the Next generation GS Junior System (NGS) setted with a detection limit of 0.05% and evaluated the clinical relevance of low prevalent mutations.

**Methods:**

NGS was performed on the plasma of 26 infected individuals who started a TDF/FTC/RPV (15 subjects) or TDF/FTC/EFV (11 subjects) cART after a routine HIV-1 drug-resistance negative test by Viroseq HIV-1 Genotyping System. Amplicon Sequencing of HIV-1 RT and PR Plate (Roche) was performed following the manufacturer's instructions. HIV-1 variants were analyzed by a specific HIV-1 tool by AVA software v. 2.7. The updated IAS resistance mutations list (March 2013) was considered for the analysis of resistance positions. Patients were followed testing viral load and immunologic parameters.

**Results:**

Twenty four males and two females with a mean age of 43 years were included. Twenty-one were nave for cART. At baseline, median HIV-RNA was 4.57 log copies/mL (range 2.15–6.57) and CD4 count 315 cells/mcL (range 16–648). In 18 patients, NGS did not detect any additional variant relevant for the selected cART compared to population genotyping. In the remaining eight patients resistance conferring mutations to part of the ongoing regimen were detected. Single mutations E138K (two cases) and M184V in three distinct patients and V90I+G190E; M184V+A98S; Y215F+V118I+T215I; L210S+T215I+F227L; and A62V+D67G+K70N+188H in the remaining five subjects. In all cases, the mutation prevalence was inferior to 5%. The mean daily reduction of VL was −3759 copies/mL in patients without NGS detected mutations and −1045 copies/mL in those with mutations. The median KM estimates for reaching an HIV-RNA blood level <50 copies/mL were 127 days and 161 days, respectively. One patient without baseline resistance selected for M184I+E138K+T215I (NGS) after four months of TDF/FTC/RPV therapy.

**Conclusions:**

NGS detected low-frequency HIV-1 variants harbouring RT drug resistance mutations that could have affected the therapy outcome. However, viral decay in an early cART phase was not affected by the presence of resistant minority variants. The low prevalence of the detected mutation, the limited effect on the combination regimen and the potency of cART components could be possible explanations of our findings. Longer follow-up and larger casuistries are needed to determine the clinical relevance of NGS in routine clinical practice and eventually define a clinically relevant mutations' prevalence.

